# Communication Research Priorities for Autism Research: Insights from a Caregiver Survey

**DOI:** 10.3390/bs16030430

**Published:** 2026-03-16

**Authors:** Taylor Huntley, Eileen Haebig

**Affiliations:** Department of Communication Sciences and Disorders, Louisiana State University, Baton Rouge, LA 70803, USA; thale5853@gmail.com

**Keywords:** autism, language, communication, research priorities, stakeholders

## Abstract

Currently, autism researchers have limited knowledge about stakeholders’ priorities for research. This raises concerns because the autism community has increasingly called for more involvement in research. The present study aimed to provide initial insight into caregiver’s priorities for research that specifically focuses on language and communication in autistic children. Seventy-three caregivers of autistic children completed an online survey with an option to participate in a follow-up feedback session (*n* = 14). Within the survey, caregivers ranked the importance of 15 communication research topics. Participants also answered questions about barriers and incentives to participating in research. Caregivers highly ranked research that focuses on learning new words, echolalia, and learning to read. Additionally, 87% indicated that they would participate in research that did not involve intervention for their child. The top barrier to participating in autism research was time, and the top incentive was if a study was virtual. Associations between priority rankings and child language skills were also explored. Word learning research was particularly important to caregivers of children who communicated using shorter utterances or through augmentative and alternative communication devices, and research that focused on abstract language was particularly important to parents of autistic children with more advanced language skills. Caregiver feedback sessions provided additional insight into the rankings of research priorities. Caregivers of autistic children value pediatric language and communication research. Many valued research topics aligned with clinical goals in therapy (e.g., learning new words) and skills that highlight less understood learning and communication processes (e.g., echolalia). We discuss how these data can guide researchers as they conduct future autism research.

## 1. Introduction

The neurodiversity movement has spurred autistic advocates to call for greater influence over the research topics that are explored and the research process. This desire aligns with the “nothing about us without us” call to action (Charlton, 1998). The neurodiversity movement allows researchers the opportunity to examine and conceptualize autism differently—such as part of human variation within a diversity of minds (Pellicano & den Houting, 2022). The neurodiversity movement has also encouraged scientists to engage in varying degrees of community-based participatory research. In this approach, researchers collaborate with the autism community. Autistic stakeholders may be fully engaged in the research process as team members or may, for example, provide input on which topics to research, the research procedures, and on data interpretation (Poulsen et al., 2022; Riccardi et al., 2023). The purpose of this research study is to take an initial step in understanding priorities for autism research that focuses on language and communication development. Thus, in the current study, we surveyed key stakeholders—caregivers of autistic children—on their opinions of what should be prioritized in research that focuses on language and communication in autistic children.

### 1.1. Stakeholder Research Priorities

Though recent changes are emerging, there has been a lack of autistic individuals and their stakeholders involved in the research process (Pellicano & den Houting, 2022). In recent years, a growing body of work documents autistic perspectives on autism research and utilizes participatory methodologies. Stakeholder input can help researchers prioritize the wants and needs of the population they are serving. However, though efforts are growing in this area (e.g., Malone, 2025), involvement of autistic children in study design is limited. Researchers tend to rely on parent input and input from autistic adults. Comprehensive research input and involvement from the autistic community is important because the differences between speaking and non-speaking autistic experiences can be contrasting. Therefore, it is important for researchers to establish relationships and include members of various autistic subgroups (den Houting et al., 2021).

Pellicano et al. (2014) took steps to examine whether research funding aligned with research topics that autism stakeholders believed should be prioritized. They identified discrepancies between topics that were desired and the federal funding of research topics at that time. Thus, they noted the importance of researchers engaging in priority-setting exercises with the autistic community. Pellicano et al.’s study involved autistic adults, autism researchers, and family members of autistic individuals. Their findings highlighted research that examined how public services could be implemented to best meet the needs of autistic people. Participants also endorsed research that focused on how best to improve life skills in autistic people and research that sought to understand how autistic individuals think and learn (Pellicano et al., 2014).

Frazier et al. (2018) also examined autism research priorities across a diverse community of stakeholders. Their online survey revealed that respondents more strongly favored applied science over basic research; however, basic science was still in the agreeable/favorable range. Four research topics were rated as very important: (1) understanding co-occurring conditions, (2) adult transition, (3) lifespan issues, and (4) health and well-being. Notably, though there was some agreement, Frazier et al. (2018) found large variability in perspectives in all aspects of autism research.

A few studies have specifically examined research priorities that focus on young autistic children. Fletcher-Watson et al. (2017) surveyed a large sample of autism stakeholders (parents, teachers, clinicians) and autistic adults on early autism research. They reported that each stakeholder group overwhelmingly favored research examining early signs of autism (Fletcher-Watson et al., 2017). Additionally, the highest ranked categories were the genetic basis of autism and early signs of autism in infants. Stakeholders also ranked the highest goals for early autism research as (1) early identification, (2) better clinical knowledge of early signs, and (3) provision of help to develop skills. It is notable that early communication delays are an early indication of autism and a feature that frequently spurs autism assessments. 

More recently, Clark and Adams (2020) surveyed research priorities of parents of autistic school-aged children. Parents were asked to consider their priorities for their children in three different settings: (1) the child’s home, (2) school, and (3) community. Results of the online questionnaire showed that the most important research priority in the home was health and well-being. In the school setting, socialization and social support were the highest ranked priorities (Clark & Adams, 2020). 

Pituch et al. (2011) designed a survey for parents of autistic children to examine treatment priorities across a variety of adaptive skills and problem behaviors. They reported that the top priorities of parents were: (1) making friends, (2) personal safety, (3) pedestrian safety skills, and (4) interacting appropriately with unfamiliar people/strangers. These findings highlight the importance of communication and social skills and interventions that can lead to improved child outcomes in these specific domains. 

Recently, Lee et al. (2025) published guiding principles and research topics to prioritize. The focus of these guiding principles was on early childhood autism therapy research. This community-engaged project identified research ideas that were specific to child-focused research. They also generated ideas for caregiver-focused research, as well as family, sibling, community, and peer-focused research, and system-focused research topics. Within child-focused research topics, the project members noted various skills to focus on: self-advocacy skills, motor skills, executive functioning skills, and language and social communication skills. They also identified child skills that have been previously used as an outcome measure for early intervention research that were believed to be harmful. For example, the team noted that interventions that aimed to reduce autistic characteristics or reduce echolalia were problematic. Though many topics were identified as important, early communication and language skills in children were included in the list of valued intervention research topics (Lee et al., 2025).

The summarized studies document that autism stakeholders care about early language and communication skills in autistic children. Indeed, the positive relationship between language and child outcomes has been documented in the literature. This relationship is discussed below to complement the stakeholder’s perceived importance of language skill development.

### 1.2. The Importance of Language for Autistic Children

As Lee et al. (2025) and others have noted (e.g., Pellicano et al., 2014), it is vital to research topics that are beneficial to the population being studied. An underlying skill that was reflected in the previously reported surveys is language (e.g., making friends, socialization). Studies have shown that one of the strongest predictors of positive life outcomes for autistic children is the acquisition of spoken language (LeGrand et al., 2021; Mayo et al., 2013; Miranda et al., 2023; Toth et al., 2006). This positive association includes the acquisition of language skills using augmentative and alternative communication methods (e.g., Gordon et al., 2011; Pope et al., 2025). During school-age years, receptive syntax, expressive syntax, and receptive vocabulary abilities serve as strong concurrent predictors of reading abilities (Åsberg Johnels et al., 2019). Given the significance of early language for an individual’s later prognosis, it is important that research provides insight into the underlying mechanisms that drive language (Toth et al., 2006). Although many studies have examined language abilities and language learning in autistic children, researchers still have an incomplete understanding of the mechanisms that drive language learning in autistic children (Arunachalam & Luyster, 2016). Considering that language is a valued subject for autism research (e.g., Fletcher-Watson et al., 2017; Lee et al., 2025) and is important for autistic children’s outcomes (e.g., Zaidman-Zait et al., 2021), it is critical to prioritize this line of research, particularly in domains of language that the autism community values. The current study aims to identify language and communication research topics that are valued by autism stakeholders.

### 1.3. Current Study

The current lack of knowledge about stakeholder priorities for research on autistic language and communication development represents a gap that should be addressed. This knowledge could aid research teams in developing more highly valued language studies and, eventually, in developing more effective language interventions and supports for autistic children and their families. The current study aims to take an initial step to address this gap in knowledge by gathering research priority rankings for language and communication research topics from caregivers of autistic children. Thus, our research questions were:

1. What developmental language domains do caregivers of autistic children consider most important for autism language research?
2. Do priorities for autism language research vary among demographic subgroups?
3. What study factors are important for participating in autism research?

## 2. Methods

### 2.1. Participants

Eighty-one caregivers of autistic children completed an online survey to address the research questions. Caregivers were recruited through community and clinic advertisements, advertisements on social media, and personal connections. Seventy-three of the 81 survey responses were based on responses of caregivers who had an autistic child who was 17 years of age or younger. Of these 73 caregivers, respondents primarily identified as mothers (*n* = 65), but the sample also included fathers (*n* = 4), parents/guardians (*n* = 3), and a grandparent (*n* = 1). The mean age of the children for whom the caregivers were advocating was 6.47 years (*SD* = 3.36, range: 2–16 years). See Table 1 for additional respondent and child characteristics. As will be described below, a qualitative component was included in the study to provide more insight into and to validate the quantitative survey data; 14 mothers agreed to participate in this supplemental component of the study.

### 2.2. Research Procedures

#### 2.2.1. Survey

The online survey was administered through Qualtrics. We first pre-screened individuals to verify that they were a caregiver of an autistic child and if the individual completing the survey was 18 years old or older. Then, we used a captcha verification to prevent bot-driven responses, and we carefully examined each submission. If the individual answered yes to both pre-screening questions, the consent form was presented. The consent form explicitly stated that there was no compensation for participating in the current study. Upon consenting to participation, the individual was brought to the study survey. The first section of survey questions included demographic information, including the number of children a caregiver has with an autism diagnosis, child’s age, child’s gender, presence of co-occurring conditions, and caregiver’s relation to the child. In the second section, caregivers were then presented with a ranking task of different language development milestones (e.g., autistic children’s first words, responding to yes/no questions, following directions).

Caregivers were asked to rank their perceived value of each language or communication topic on a scale from 0 (not at all important) to 100 (very important). Afterwards, caregivers were asked to list their top three most important research priorities as first, second, and third using separate drop-down boxes. In the next section, caregivers were asked to describe their child’s language abilities, consisting of a prompt to select a language stage that best fits with their child’s current language abilities, and to identify their child’s strongest language ability and greatest language difficulty. We also asked caregivers to list barriers to participation in research and to identify the types of research that they would be willing to participate in. In addition, we asked the caregivers to select, from a list of options, things that would encourage participation in research. Lastly, we asked if the caregiver would be willing to participate in research that does not include treatment or intervention for their child. Additionally, at the end of the survey, we provided an opportunity for the participants to type any comments that they wished to share with the research team. We provide the full survey in the Supplementary Materials.

#### 2.2.2. Feedback Session

In order to validate and gain further insight into the quantitative survey data, at the end of the survey caregivers had an option to indicate whether they would like to participate in a follow-up video or phone call to further discuss their survey responses. If a participant indicated that they were interested in participating in the follow-up feedback session, a research assistant emailed the individual to schedule a time to meet. The feedback session was conducted via a Zoom video call and was led by the first author or a lab researcher. The feedback session consisted of pre-determined short open-ended questions asking the caregiver to expand on personal barriers to participating in research, which autism research studies they would be willing to participate in, and their reasons behind selecting their top three research priorities. Caregivers were also asked if there were language topics that they did not choose as their top research priorities that were highly important to them, or if there were more language topics that they think are important to research that were not included in the survey. Additionally, caregivers were asked if there were any other language topics that should be researched that were not listed in the interview. At the conclusion of the Zoom call, caregivers were asked if they had anything else they would like to state about the language topics or about the study in general. Each Zoom call was intended to last approximately 15 min; the durations ranged from 8 min to 32 min, with a mean of 16.4 min. The protocol for the feedback session can also be found in Supplementary Materials.

Upon completion of the Zoom video call, the recording and the automated transcript were saved to the lab server. To assess this supplemental insight and validation of the quantitative survey data, all transcripts were reviewed to categorize themes. All categories/themes were documented and then organized by frequency; this process was completed by two research assistants separately. When identifying themes, the coders created a file with each theme noted and the number of interviewees who spoke about each of the themes. Then, the two coders met to discuss the themes that were identified and created a summary document with examples of direct quotes from the feedback sessions to support the assurance of the identified themes. These themes were also further discussed with the first author of the study, who conducted the majority of the post-survey research sessions and who had created an initial summary of the topics that the parents discussed.

### 2.3. Positionality and Community Involvement Statements

The authors of the current study do not identify as autistic individuals. The authors are clinical researchers who have worked with autistic children in clinical and research contexts. The broader research team members who were involved in data coding and data discussions included neurodiverse individuals (autistic individuals and individuals with ADHD) and individuals who were autistic stakeholders (e.g., student clinicians who worked with autistic children and family members of autistic individuals). The survey was developed following careful examination of the available surveys from the articles that were previously reviewed in this document. Additionally, the child language and communication topics that were included in the survey were selected based on the authors’ clinical experiences and research knowledge. Feedback about the survey was also sought from an autistic speech-language pathologist who at the time was working in a pediatric clinic serving autistic and non-autistic children and a speech-language pathologist who is also the mother of an autistic child with limited spoken language abilities. We are grateful for this broader involvement given the authors’ positionality.

### 2.4. Data Analysis Plan

Descriptive statistics were gathered from the survey responses to provide an overview of demographic characteristics of the caregivers who completed the survey (i.e., age, race, ethnicity, and educational level) and the children (i.e., age, gender, language abilities). To address the first research question, the mean and standard deviation of the caregiver rankings of each developmental language domain were calculated. We also tallied the first, second, and third most important research topic that was selected for the categorical ranking-of-importance questions. Additionally, we identified the most common themes from the feedback sessions related to the rationale for selecting the parent’s top research priorities.

To address our second research question, survey responses were sorted into subgroups by child gender and language abilities. The mean rankings for each language stage were descriptively compared between subgroups. Additionally, we conducted Pearson’s correlations to examine associations between child age and language priority rankings. Spearman’s rank-order correlations were conducted to examine associations between child language level and caregiver’s language priority rankings for each language topic. The following scheme was used to rank child language abilities: A score of 1 was assigned to non-speaking children, children who primarily use gestures, and children who primarily use AAC devices to communicate. A score of 2 was assigned to children who produce a few words, one at a time. A score of 3 was assigned to children who combine two words; a score of 4 was assigned to children who use echolalia as a primary means of communication. A score of 5 was assigned for children who speak in simple sentences (3–4 words), and a score of 6 was assigned for children who produce complex sentences to convey abstract thoughts and ideas.

When addressing the third research question, we tallied the barriers and incentives to participating in research that caregivers reported in the survey. We also calculated the percentage of caregivers who indicated that they would be willing to participate in research that did not include intervention for their child.

### 2.5. Data Availability

De-identified data may be requested, and reasonable requests will be approved.

### 2.6. Ethical Considerations

All study procedures were approved by Louisiana State University’s IRB (IRBAM-22-0922). All participants completed an electronic consent form prior to beginning the survey.

### 2.7. Generative AI Statement

Generative artificial intelligence (GenAI) was not used to create this manuscript, and it was not used in any part of the research process (e.g., GenAI was not used to create the research survey, to collect data, to process data, or to conduct analyses).

## 3. Results

### 3.1. RQ1: What Developmental Language Domains Are the Most Important to Caregivers for Pediatric Autism Language Research?

**Figure 1:**
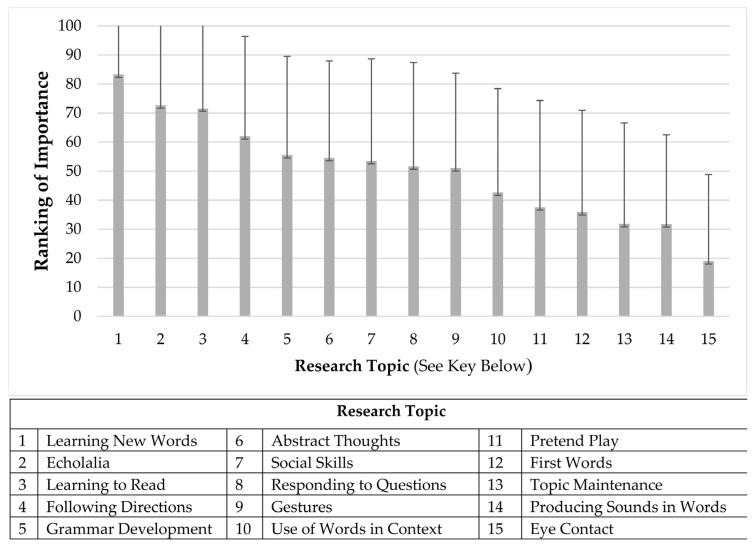
Overall caregiver judgments of research priorities.

We asked caregivers to rank the research priorities using a sliding scale (0–100) and later to use a drop-down box to identify their top three research priorities. The scale ratings revealed that caregivers most highly ranked research that focused on how autistic children learn new words (*M* = 83.27, *SD* = 27.38). The second-highest ranking was for research focusing on echolalia (*M* = 72.68, *SD* = 32.24), and the third was learning to read (*M* = 71.60, *SD* = 31.71). Each of the 15 language research topics had at least one caregiver who scored the topic as 0 (not at all important) and at least one caregiver who scored the topic as 100 (very important). This contributes to the large standard deviation of each language topic. See Figure 1 for mean rankings for each language priority.

#### 3.1.1. Top Three Research Priorities Ranking

After participants used the sliding scale to rank each language topic, the caregivers used three drop-down menus that included each of the 15 topics. They then selected their top three language research topics through this categorical method. Overall, participants ranked the following as their top priorities: (1) how autistic children learn new words (36% of all first choices), (2) echolalia (16% of all second choices), and (3) following directions (i.e., sentence comprehension; 14% of all third choices). When all responses of the first, second, and third topics ranked most important were collapsed into one list, how autistic children learn new words was the overall most important research topic (selected within the top three priorities by 20% of the caregivers), followed by echolalia (18%), and then following directions (11%). This is consistent with the item-level rankings, as these three priorities were in the top four means that were presented in Figure 1. Surprisingly, the learning to read topic was ranked as third highest on the overall item-level ranking but was not listed as a top research priority in the section that asked caregivers to select their top three most important topics.

#### 3.1.2. Parent Feedback About Research Priorities

During the follow-up feedback session with the 14 mothers who participated in this additional component of the study, a common rationale for selecting the top research priorities was that the topic was relevant to their child’s current language stage or language growth goals. Many agreed that language research should be a top priority; one mother specifically stated, “if we’re going to do research in autism and autistic kids—and this is what’s going to help them the most—their communication has to be the top priority.”

Caregivers also stated that they selected research topics that they believed were important for their child’s future success and safety, and because they wished to improve their interactions with their child (most related to the social skills topic, *n* = 2). Additionally, 29% (*n* = 4) of the mothers discussed the need for future studies to address autistic self-generated language through a neuro-affirming lens, with an emphasis on echolalia and gestalt language processing, sometimes specifically noting the Natural Language Acquisition Protocol (Blanc et al., 2023). Neuro-affirming approaches aim to promote or focus on strengths that individuals have when conducting research studies, including intervention research (Lerner et al., 2023). An example could include research studies that explore verbal working memory strengths associated with children who engage in echolalia. Another example could include research interventions that aim to build on echolalic scripting abilities in autistic children as a method to enhance broader language skills. One mother stated: “so many autistic kids, including my son, use echolalia—delayed echolalia—a lot to communicate, and I think these parents and/or caregivers and the field supporting these kiddos would like to do well [by] explor[ing] this area more.”

### 3.2. RQ2: Do Priorities for Pediatric Autism Language Research Vary Among Demographic Subgroups?

#### 3.2.1. Language Stage

The largest variation when completing inter-group comparisons was associated with the child’s language stage. This can be seen through comparing these sub-groups to each other. For example, the caregivers of autistic children who speak primarily using echolalia ranked echolalia as the most important language research topic on the survey. Overall, with each group of language abilities, these caregivers rated different research topics as their first, second, and third most important than compared the other groups. We present these results in Table 2.

#### 3.2.2. Child Gender

There were 50 male, 20 female, and 3 transgender or nonbinary children whose caregivers completed the survey. The mean priority ranking for each topic within each gender subgroup was descriptively analyzed. The differences between each research priority from these sub-groups were less than 15.55 points, suggesting substantial overlap in research priorities between genders.

#### 3.2.3. Research Priority Correlations

Pearson’s correlations were conducted to examine the association between child chronological age and caregiver ranking of language research priorities. None of the rankings were significantly correlated with chronological age (*p*s > 0.14). To determine if there were any associations between child language ability and caregiver rankings, we conducted Spearman’s rank-order correlations. Two associations were significantly correlated. The shorter the spoken utterance length of the child or if the child was an AAC user, the higher caregivers ranked learning new words (ρ = −0.241, *p* = 0.046). Additionally, children who produced longer spoken utterances tended to have caregivers who highly ranked the understanding of abstract thoughts topic (ρ = 0.304, *p* = 0.011). All other correlations were non-significant (*p*s > 0.15).

### 3.3. RQ3: What Study Factors Are Important for Participating in Autism Research?

The last portion of the survey was dedicated to understanding factors that could impact study participation. Some caregivers chose not to respond to each question or indicated that the question was not applicable. With these reduced sample sizes, we report the barriers that may keep caregivers or their children from participating in research (*n* = 62), what may encourage research participation (*n* = 66), and if caregivers would allow their child to participate in research that did not involve treatment (*n* = 68).

#### 3.3.1. Barriers

The top barrier caregivers listed for participating in research was time (*n* = 19; 31%). The second-highest response was their child’s wellbeing (*n* = 9; 15%). For example, some parents had concerns that a study’s protocol may be too taxing or invasive for their child (e.g., bloodwork involved with genetic research or developmental assessments that may cause the child to become overwhelmed). The third-highest response was concerns regarding researchers’ motives (*n* = 6; 10%). Themes that surfaced during the follow-up feedback session with the 14 mothers included: 1. concerns about researchers using traditional research approaches or examining topics including applied behavioral analysis, medical experiments, and some clinical experiments (*n* = 6); 2. concerns about different priorities between researchers and the community with concerns that researchers lack true understanding of autism, the community, and autistic communication style (*n* = 4); and 3. lack of time (*n* = 3).

#### 3.3.2. Caregiver Priorities and Preferences for Participation

Caregivers were prompted to identify the types of research they would be motivated to participate in using a text box. The highest reasons to participate were: if the study was an online study or at a specific location (*n* = 43, 65%), if the study was on a preferred research topic (*n* = 17, 26%), and if the study was not invasive or did not involve neuroscience (*n* = 13, 20%). When caregivers were asked to select from a list of options of possible incentives for research participation, the top incentive was if the study was online (*n* = 42, 62%), followed by length of the study (*n* = 37, 54%, with a preference for short studies), payment/compensation (*n* = 36, 53%), and location of the study (*n* = 27, 40%). In the feedback sessions, the mothers overwhelmingly reported virtual studies were more accessible for them due to time and scheduling constraints. Lastly, when asked if the caregiver would be willing to participate in research that did not involve treatment for their child, 59 of the 68 survey responses (87%) indicated that they would participate.

## 4. Discussion

With increasing calls for community involvement in autism research, it is critical that researchers consider autistic stakeholders’ thoughts and preferences. Given the general support for language research in the previous surveys that sought stakeholder input on research priorities (e.g., Clark & Adams, 2020; Lee et al., 2025; Pituch et al., 2011), and the well-established association between language skills and autistic children’s life outcomes, it is reasonable to believe that researchers will conduct language studies. The current study added increasing support and direction for autism research that focuses on language and communication development. After reviewing our data (from the priority rankings, subgroup comparisons, and supplemental post-survey feedback sessions), it is clear that caregivers value autism research that focuses on language and communication. Additionally, we found that caregivers’ language research priorities are typically based on their child’s current language level and needs. Despite the confirmation that caregivers value this research, our studied identified concerns that caregivers had about participating as well (e.g., time-intensive studies, procedures used or questions based on certain theoretical domains, and procedures that limited their child’s ability to demonstrate their true knowledge and language learning abilities).

Research that focused on examining how autistic children learn new words was the highest ranked topic. Fortunately, this topic has not been ignored in the existing research literature (e.g., Arunachalam et al., 2024; Choi et al., 2016; Gladfelter & Goffman, 2018; Haebig et al., 2017; Henderson et al., 2014; Kirkorian et al., 2016; Luyster & Arunachalam, 2020; McDuffie et al., 2006; Venker et al., 2016). Future word learning studies would be valued as well. Notably, vocabulary knowledge is a key predictor of concurrent and later reading abilities in autistic children (Åsberg Johnels et al., 2019; Westerveld et al., 2017). Reading research was also highly rated by caregivers, which could possibly indicate that research projects that include both topics could be well-motivated and highly valued by the autistic community. Though this topic has not been overlooked in the prior literature (e.g., Bullen et al., 2022; Caron et al., 2018; Davidson et al., 2018; Dynia et al., 2019; Henbest & Apel, 2024), this is a less studied domain.

Additional topics that were highly valued by the caregivers included echolalia, responding to questions, following directions (i.e., sentence comprehension), and grammar development. These highly ranked topics have not been completely ignored either (e.g., Cohn et al., 2024; Cohn & Harrison, 2025; Daar et al., 2015; Kover et al., 2014; Sterling, 2018). However, it is important to place emphasis on these research topics in future studies and to consider how studies can incorporate the feedback that the caregivers provided related to barriers and incentives for participating in research. 

One notable theme that was observed in the caregiver comments and rankings, was echolalia research. Though research as far as 40 years ago has noted the significance of echolalia for communication (e.g., Prizant, 1983; Prizant & Duchan, 1981), we still have a limited understanding of this topic. Existing data-based intervention research that focuses on echolalia has overwhelmingly come from one perspective and aimed to reduce or extinguish echolalia (Dinello & Gladfelter, 2025). Furthermore, the vast majority of these studies did not result in increases in self-generated spoken communication despite reductions in instances of echolalia (Dinello & Gladfelter, 2025). Additionally, one clinical intervention focusing on echolalia has recently received a great deal of attention but is not currently supported by data-based research (i.e., natural language acquisition protocol; see the following for a discussion about this issue: Bryant et al., 2024; Hutchins et al., 2024; Lorang et al., 2025; Venker & Lorang, 2024). Thus, future research in echolalia would be particularly valued by stakeholders, including clinicians and autistic adults (see Lee et al., 2025). 

Compared to the extant literature, the current study yields some similar results in regard to stakeholder attitudes. Clark and Adams (2020) found that often caregivers discussed that the research priorities that were not ranked as high were not less important, but rather that these topics have already been researched. Within our current study, caregivers noted that they ranked priorities based on their family’s needs at the time of the survey. This emphasis on the family’s current needs highlights the importance of gathering input from caregivers of autistic children. Since caregivers are the ones who must consent to the research projects and coordinate their child’s and family’s schedules to find time to participate, caregiver feedback gathered from individuals who have autistic children at developmental points that overlap with potential research studies is valuable. It may be difficult for caregivers of autistic children who are already grown or autistic adults who do not currently have children to provide input, although this input still holds value. 

In the current study, consideration of the child’s expressive language abilities provided added insight about differences in research priorities. Specifically, our correlational analyses revealed that caregivers were more likely to highly value word learning research if their child was in the earlier stages of language development or communicated primarily through alternative or augmentative communication. Conversely, caregivers were more likely to highly value research that focused on how autistic children understand abstract thoughts if their child was in more advanced stages of language development. Surprisingly, although Fletcher-Watson et al. (2017) identified early language as an important research priority for autism research, our current findings revealed that research examining autistic children’s first words was ranked as the fourth-lowest priority. Given these results in the other studies, we would have expected this specific topic to be ranked higher by the caregivers who participated in the current study. When considering this mismatch, it is important to remember that the current study included language skills that extend beyond the early language acquisition phase. The current survey was able to give caregivers a say in a larger variety of language and communication topics. Furthermore, research topics that received lower priority rankings may still hold clinical and theoretical value; however, researchers should consider their study aims and procedures carefully to optimally design studies that will be meaningful both theoretically and clinically. The use of autism advisory boards or community-based participatory research methods could positively impact research projects and enhance a study’s impact on both the research community and autism community.

Another aim of the current study was to assess aspects of the research that caregivers may find important and to influence participation. In the current survey, 87% of caregivers reported that they would be willing to participate in research that did not involve treatment for the child. Although previous studies indicate that applied research studies are important to the autistic community (e.g., Clark & Adams, 2020), the current study demonstrates caregivers’ willingness to participate even when intervention is not a component of a research study. This is promising because several valued research topics can be addressed without intervention being a study component (e.g., echolalia, grammar development, following directions/sentence comprehension). Furthermore, it is important to note that intervention studies tend to be quite time-intensive, and this feature was identified as a barrier to participating in studies in our data.

Several caregivers also indicated that the location of the research, and particularly online studies, impacted participation. Online studies were greatly preferred. This may help shape the way future researchers design their studies; however, this could potentially create barriers for researchers to overcome. It may be difficult for researchers to optimally measure their research questions because many clinical assessments and research tasks require object manipulation or pointing, which would be difficult or impossible on an online platform (especially for young children and children with high support needs). Additionally, some experimental paradigms are not possible to use in online tasks, and some data cannot be collected online (e.g., neuroscience studies). Furthermore, difficulties with stable internet access may impede study participation, particularly for families that are economically disadvantaged or who live in rural areas.

### Limitations

Although this study added missing information in the current literature, namely information about research priority preferences regarding specific language and communication topics, there are limitations that must be noted. First, participants were not compensated for their involvement. We acknowledge that the lack of compensation may have introduced sampling bias, potentially limiting the diversity of our sample, both in demographic categories and possibly in diversity of perspectives. Second, this study included a relatively small number of participants and somewhat limited diversity in racial and ethnic backgrounds and other demographic characteristics. We were unable to compare additional subgroups due to this. Third, we did not ask the caregivers who completed the survey to disclose whether they also identified as autistic or had an autism diagnosis. Previous work has demonstrated that rankings of research priorities sometimes differ between autistic adults and non-autistic stakeholders (Fletcher-Watson et al., 2017). As such, it would have been interesting to examine whether there were differences between autist and non-autistic caregivers in priority rankings for the research topics that we examined. Fourth, we also did not ask the caregivers to describe their thoughts about autism broadly or the degree of acceptance of the unique characteristics of their autistic child. A caregiver’s perspective about autism could be associated with the selection of top research topic priorities (e.g., a priority for clinical researchers identifying a cure for autism vs. a priority for neuro-affirming practices). Fifth, although we were able to gain some qualitative data to supplement our quantitative data, the post-survey feedback sessions were short, and we were not able to conduct a comprehensive qualitative analysis.

## 5. Conclusions

Autism language researchers can benefit from these results. The current study revealed that caregivers of autistic children value autism research that focuses on language and communication development. Furthermore, research that focuses on how autistic children learn new words, follow directions, learn to read, and respond to questions, and research on echolalia were consistently ranked as highly valued overall and should therefore receive more emphasis in future research. Additionally, we identified some instances of variation in caregiver rankings according to child language stage, indicating that researchers need to consider the child’s language ability when identifying specific research topics to pursue. Lastly, we identified barriers and incentives to research participation that could improve research protocols and therefore, study participation and appreciation of findings.

## Figures and Tables

**Table 1:** Participant characteristics.

Participant Characteristics (*N* = 73)			
Adult Characteristics		Child Characteristics	
Adult Ethnicity	Non-Hispanic (*n* = 63) Hispanic (*n* = 7) Prefer Not to Answer (*n* = 3)	Child Age	*M* = 6.47 *SD* = 3.36 Range: 2–16 years
Adult Race	White (*n* = 53) Black or African American (*n* = 4) American Indian or Alaska Native (*n* = 2) Multiple Races (*n* = 3) Prefer Not to Answer (*n* = 4)	Child Gender	Female *n* = 20 Male *n* = 50 Transgender or nonbinary *n* = 3
Highest Level of Education	Graduate/Professional degree (*n* = 39) Bachelor’s degree (*n* = 22) Associate’s degree (*n* = 5) High School Degree/GED (*n* = 7)	Child Language	Non-Speaking *n* = 13 First Words Stage *n* = 4 Word Combination Stage *n* = 6 Primarily Produces Echolalic Language *n* = 12 Simple Sentences Stage *n* = 16 Complex Sentences Stage *n* = 18
Region of the United States	Southeast (*n* = 30) Southwest (*n* = 9) West (*n* = 12) Northeast (*n* = 15 Midwest (*n* = 5) Prefer Not to Answer (*n* = 2)		

**Table 2:** Caregiver judgments of research priorities based on child’s language level.

	Non-Speaking (Gestures & AAC, *n* = 13)	First Words Stage (Few Words, One at a Time, *n* = 4)	Word Combination Stage (Two Words, *n* = 6)	Primarily Produces Echolalic Language (*n* = 12)	Simple Sentences Stage (3–4 Words, *n* = 16)	Complex Sentences Stage (*n* = 18)
Top Priority	How autistic children learn new words (*M* = 91.5)	Social skills (*M* = 77.0)	How autistic children learn new words (*M* = 91.8)	Echolalia (*M* = 85.8)	How autistic children learn new words (*M* = 92.8)	How autistic children learn new words (*M* = 70.7)
2nd Highest Priority	Learning how to read (*M* = 79.9)	Learning how to read (*M* = 72.5)	Responding to questions (*M* = 83.2)	How autistic children learn new words (*M* = 81.9)	Learning how to read (*M* = 78.6)	Learning how to read (*M* = 66.7)
3rd Highest Priority	Echolalia (*M* = 70.2)	Participating in pretend play (*M* = 69.3)	Following directions (*M* = 75.0)	Learning how to read/Grammar (tie) (*M* = 57.8)	Echolalia (*M* = 78.4)	Understanding abstract thoughts (*M* = 64.7)

## Data Availability

Deidentified quantitative data are available at https://github.com/ehaebig/research_priority_ratings (accessed on 13 January 2026).

## Supplementary Materials

The following supporting information can be downloaded at: https://www.mdpi.com/article/10.3390/bs16030430/s1.
