# Supplementary material for: Communication Research Priorities for Autism Research: Insights from a Caregiver Survey
Source: Behav Sci (Basel). 2026 Mar 16;16(3):430. doi: 10.3390/bs16030430 (PMC13023947; doi:10.3390/bs16030430)
Supplement: Supplementary file 1 [file behavsci-16-00430-s001.zip › behavsci-4125811-supplementary.pdf]

Supplementary Materials

Prescreener

Do you have one or more children who have an autism spectrum diagnosis?

Yes

No

Are you 18-years-old or older?

Yes

No

Complete the Catpcha below to confirm that you are not a robot.

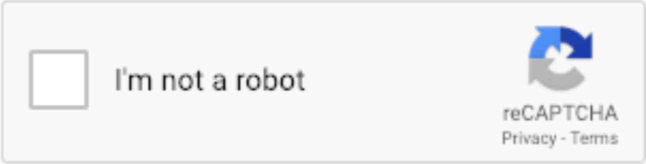

Consent

Study Title: Research Priorities for Child Language Research Focusing on Autism

The purpose of this research is to understand what parents of autistic children want researchers to focus their efforts on studying when examining child language in autistic children.

What does this study involve?

This study will consist of one online survey that will last 5-10 minutes. You will be asked questions about your opinions about priorities for research that focuses on language development in autistic children. Also, you will be asked to answer basic questions about your child, like your child's age and language abilities, and basic demographic questions about yourself.

You will also be asked if you would like to participate in a follow-up Zoom call to further discuss your opinions about priorities for research focusing on language development in autistic children. If you also choose to participate in the interview, the call will last approximately 15-20 minutes and will be video or audio recorded.

Participant Inclusion: You must be at least 18 years old and have at least one child who has been diagnosed with autism spectrum disorder.

Participant Exclusion: You are ineligible to participate in this study if you are under 18 years of age or do not have at least one child who has been diagnosed with autism spectrum disorder.

Risks: There are no risks that are beyond minimal risks associated with participating in any research study. There is a risk of an inadvertent release of sensitive information that you may choose to provide about yourself or your child; however, every effort will be made to maintain the confidentiality of your study records. Files will be kept in secure cabinets to which only the investigator has access and/or saved in secure digital storage space (e.g., lab server).

Participants who choose to participate in the Zoom call will be video or audio

recorded and the files will be saved to an LSU lab server and the LSU OneDrive or LSU Box cloud-based server, which are secure data platforms. These recordings will be deleted within 24 months of the recording; however, the de-identified transcript of the interview will be kept indefinitely.

Benefits: There are no direct benefits from participating in this experiment; however, we hope to learn things that will help improve our understanding of what families want researchers to focus on when conducing autism research that focuses on language. We hope that this knowledge will help others in the future.

Compensation: There is no compensation associated with the current survey study.

Performance Site: Online

Investigators: The following investigators are available for questions about this study: Principal Investigator: Dr. Eileen Haebig, PhD E-mail: ehaebig1@lsu.edu; Graduate students: Taylor Hale, thale6@lsu.edu; Fatema Mitu, fmitu1@lsu.edu; Caprielle Priola, cpriol1@lsu.edu; Jesica Sykes, jsykes8@lsu.edu; Undergraduate students: Alyssa Schell, and Naomi Tomiyama - (225) 578-3933.

Right to Refuse: Your participation in this study is completely voluntary. You may choose not to participate, and you may withdraw from the study at any time without penalty. You may skip over questions or procedures, or you may withdraw by informing the researcher that you no longer wish to participate (no questions will be asked). Your decision to participate, decline, or withdraw participation will have no effect on your status at or relationship with Louisiana State University.

Privacy: Every effort will be made to maintain the confidentiality of your study records. Your responses will be assigned a code number that is not linked to your name or other identifying information. All signed electronic consent forms will be stored in a manner that can only be accessed by the researchers. Please be aware

that no form of communication is 100% secure. Employers or others may be able to track the information you type into a computer. Results of the study may be published, but no names or identifying information will be included in the publication. Subject identity will remain confidential unless disclosure is required by law. Your responses to questionnaires and computerized tasks will be stored indefinitely. Your permission to use the data in future research studies will not be obtained, since the data will not contain any identifiers. Data from this project may be shared with other researchers or made publicly available for scientists to use in future work. If data are shared, it will be shared with your encrypted study identification number and not with any of your personal information. By participating in this study, you agree to allow the researchers to use the data for future, as yet unknown research studies.

This study has been approved by the LSU IRB. For questions concerning participant rights, please contact the IRB Chair, Dr. Alex Cohen, 225-578-8692, or irb@lsu.edu

By continuing this survey, you are giving consent to participate in this study.

Your information collected as a part of this research, with identifiers removed, may be used or distributed for future research.

IRBAM-22-0922

Do you consent to participation in this study?

Yes

No

Default Question Block

You have agreed to participate in a survey that is gathering information about priorities for autism research that focuses on child language. Please complete the following questions to the best of our knowledge.

Note: Many people from the autism community have indicated that they prefer identity-first language (i.e., autistic child) over person-first language (i.e., child with autism). We know that this is not everyone's preference; however, this is the reason that we have chosen to use identity-first language in the current survey.

This survey has been reviewed by multiple autism stakeholders including: The LSU Language NeT Lab, an autistic speech-language pathologist, and a mother of an autistic child.

How many autistic children do you have?

If you have multiple children with an autism diagnosis, please respond to the survey questions for only one of your children.

1
2
3
4+

How old is your autistic child?

What is your child's gender?

Male

Female

Non-binary

Other

Does your child have any co-occurring conditions? If so, please list them (e.g., ADHD, apraxia, anxiety).

What is your relation to your child?

mother

father

grandparent

guardian

other

What is your ethnicity?

Hispanic, Latino/a, or Spanish origin

Not Hispanic, Latino/a, or Spanish Origin

Prefer Not to Answer

What is your race? (select all that apply)

American Indian or Alaska Native

Asian

Black or African American

Native Hawaiian or Other Pacific Islander

White

Prefer Not to Answer

What is your highest level of education?

Some High School

High School Degree/GED

Some College

Associate's Degree

Bachelor's Degree

Graduate/Professional degree

Prefer Not to Answer

What region of the United States do you live in?

Northeast

Southeast

Midwest

West

Southwest

Which of the following do you think is the most important topic to research for autistic children?
Please rank level of importance using the sliding scale under each item with 0 being not at all important and 100 being very important.

	0	10	20	30	40	50	60	70	80	90	100
1. Autistic children’s first words											
2. Child's ability to respond to questions											
3. Child gestures											
4. How autistic children learn new words											
5. Making eye contact											
6. Correctly producing sounds in words											
7. Following directions (language understanding skills)											
8. Development of grammatical language											
9. Social skill development (e.g., Interacting with other children)											
10. Staying on topic in conversations											
11. Using words in correct contexts											
12. Participation in pretend play											
13. Learning to read											

14. Understanding abstract thoughts

15. Echolalia (e.g., repeating previously heard words or phrases)

Of the previous list, what is the most important research topic that you think researchers should focus on?

First (most important topic)

What is the second most important research topic that you think researchers should focus on?

Second most important topic

What is the third most important research topic that you think researchers should focus on?

Third most important topic

Describe your autistic child's language abilities/ how they communicate with parents or other adults.

Nonverbal

Produces a few words, one at a time

Combines 2 words when communicating

Speaks in simple sentences (3-4 words)

Produces sentences with complex thoughts/ideas

Primarily uses echolalia to communicate

Primarily communicates through gestures and/or communication devices (AAC)

What would you say is your autistic child's strongest language ability?

What would you say is your autistic child's greatest language difficulty?

What are barriers that may keep you from participating in autism research?

What autism research would you be willing to participate in?
For example:

- a specific research topic; schedule: one study session vs. multiple study sessions
- style: in-person vs. online
- behavioral vs. brain studies (e.g., talking tasks vs. MRI or EEG studies).

What are some things that would **encourage** you to participate in research? (Select all that apply.)

Payment compensation for participating

Duration - short studies

Duration - multiple visits

Online study

Treatment/intervention study

Location

Other

Would you be willing to have your autistic child participate in a research study that does not include treatment/intervention?

Yes

No

Would you be willing to have a 15-minute zoom meeting to expand on any thoughts related to this survey?

Yes

No

If yes to the previous question, please provide your email address.

Are there any final thoughts or comments you would like to share?

Powered by Qualtrics

Zoom Interview Protocol

The research assistant will initiate the Zoom call and record the call to the Zoom cloud. During the interview, the research assistant will ask the questions that are listed on the Zoom call interview form while noting the original survey responses.

a. Thank you again for agreeing to meet. In this short meeting, I am going to ask you to expand on the things that you noted in the survey.
b. First, in your survey response, you noted that a personal barrier to participating in research is XXX. Can you expand on this?
c. Can you expand on your answer about which autism research you would be willing to participate in? On the survey, you stated that you would be willing to participate in studies that XXX.
d. The top three research priorities that you selected were XXX. Can you explain in further detail why you chose these three priorities?
e. Were there any language topics that you did not list in your top three that you think are important? Why?
f. Do you have anything that you would like to further explain regarding the language research topics?
g. Do you think there are any other language topics that should be researched that were not listed?
h. Do you have any other thoughts you would like to share or questions that you would like to ask regarding this survey?
i. Thank you for your time.
